# An Assessment and Comparison of Root Resorption With Two Different Corticotomy Techniques During Anterior Teeth Retraction: A Split Mouth Prospective Clinical Study

**DOI:** 10.7759/cureus.33431

**Published:** 2023-01-05

**Authors:** Karthik J Kabbur, Shruthi Kamaraj, Ramesh B, Hemanth M, Aravind M, Jayanth NR

**Affiliations:** 1 Orthodontics and Dentofacial Orthopaedics, Dayananda Sagar College of Dental Sciences, Bengaluru, IND; 2 Orthodontics and Dentofacial Orthopedics, Dayananda Sagar College of Dental Sciences, Bengaluru, IND

**Keywords:** tooth movement, bimaxillary protrusion, cbct, root resorption, corticotmy

## Abstract

Aim

The aim of this prospective split-mouth clinical study is to determine the apical root resorption of anterior teeth in patients with two different methods of corticotomy namely indentation and vertical corticotomy techniques.

Methods

Sixteen adult patients with bimaxillary protrusion requiring the need for extraction of the first premolars were included in the study. For each patient, the particular method of corticotomy technique was assigned randomly along with the side and the procedure was performed following which retraction forces were applied with the help of a closed coil Niti spring. The root resorption was recorded during the stage of space closure for which two cone beam computed tomography (CBCT) were taken, one before the retraction forces could be applied (T1) and one CBCT (T2) after the space closure had occurred. The apical root resorption was assessed and analyzed both linearly and volumetrically using the CBCT taken at T1 and T2.

Results

There was root resorption present after the space closure was complete in both the maxillary and mandibular arches. The amount of root resorption that occurred in the indentation technique was slightly lesser as compared to the vertical corticotomy technique.

Conclusion

Indentation corticotomy cuts for accelerated tooth movement are considered to be a much safer, more effective method, lesser invasive to the surrounding tissues, technique sensitive, and good regional acceleratory phenomenon (RAP), with a Rapid healing process compared to the vertical corticotomy technique.

## Introduction

Malocclusion can be defined as an occlusion in which there is a mal relationship between the arches in any of the planes of spaces or in which there are anomalies in tooth position beyond normal limits [1]. Malocclusion has been shown to affect periodontal health, increase the prevalence of dental caries, and cause temporomandibular joint problems [1].

Fixed mechanotherapy is the most commonly used procedure to correct these malocclusions. During fixed orthodontic tooth movement there are a series of chemical, molecular, and histological changes that occur in the tissue and this process involves physiologic alveolar bone adaptation to mechanical strains with minor reversible injury to the periodontium. In conventional orthodontic treatment protocol, the patient is treated for a period of two years wherein the treatment is done in three phases including leveling and aligning, space closure, and then finishing phase [1]. Space closure is one of the most challenging processes in orthodontics and when using nickel-titanium springs, a rate of 1-1.2 mm space closure per month is seen [2].

Corticotomy-assisted orthodontic treatment is an established and efficient orthodontic technique that has recently been studied in a number of publications. It has gradually gained popularity as an adjunct treatment option for the orthodontic treatment of adults. It involves selective alveolar decortication in the form of decortication lines and dots performed around the teeth that are to be moved. It is done to induce a state of increased tissue turnover and transient osteopenia, which is followed by a faster rate of orthodontic tooth movement [3]. Accelerated tooth movement has three main approaches:

1. Local administration of chemical substances 

2. Physical stimulation (i.e., electrical current or magnets), and

3. Surgery (i.e., alveolar corticotomy, compression, or distraction) [4].

Corticotomy-assisted orthodontic tooth movement is one of the surgical methods of accelerating orthodontic tooth movement for space closure. Corticotomy-assisted orthodontics has advantages such as less root resorption, due to decreased resistance of cortical bone, more bone surrounding teeth, due to the addition of bone graft, and less need for extraoral appliances and orthognathic procedures [4]. With an increase in age, the density of the adult bone increases hence during tooth movement there is bound to be more resistance offered to the tooth root by the bone [5]. Adult orthodontics has become increasingly ingrained with biologic processes underpinning tooth movement slower in adults, hence the requirement of accelerated treatment duration [6]. A number of attempts have been made to create different approaches clinically in order to achieve quicker results to comply with patient needs without reducing the efficiency of the treatment [7,8].

There are various iatrogenic effects of orthodontic therapy, the major being external apical root resorption. External apical root resorption is the reduction in the root structure involving the apices of the root and is a common phenomenon in orthodontic treatment in the modern world [5]. It is a multi-factorial process that may lead to tooth loss.

To assess root resorption there are many radiographic aids that can be used which include the intraoral periapical radiograph (IOPAR), orthopantomogram (OPG), and lateral cephlograms. The newer aids including cone beam computed tomography and CT are now used to better analyze the root resorption [5].

Hence in this study, we will be assessing the root resorption in the two most commonly used corticotomy methods namely indentation corticotomy and vertical corticotomy using a CBCT which can be a better aid to evaluate of the root structure. The study being a split-mouth study will also reduce the amount of bias involved as the subjects act as their own controls, thus eliminating inter-subject variability and heterogeneity thereby increasing the power of the study. This study is designed with a prospective randomized split-mouth clinical trial.

## Materials and methods

The study consists of 16 samples which were determined using GPower software v3.1.9.2, with an effective size of 0.307. chosen from the patients reporting to the Department of Orthodontics and Dentofacial Orthopedics from Dayananda Sagar College of Dental Sciences who require orthodontic treatment.

Inclusion criteria included a) patients requiring extraction of premolars for orthodontic treatment (bimaxillary malocclusion), b) patients in whom the treatment plan was to undergo corticotomy-assisted orthodontic treatment. c) patients aged between 18-35 years. Exclusion criteria included a) patients with a history of bone disorders, systemic diseases, pregnancy, and on steroid therapy, and b) patients with poor periodontal health.

**Figure 1:**
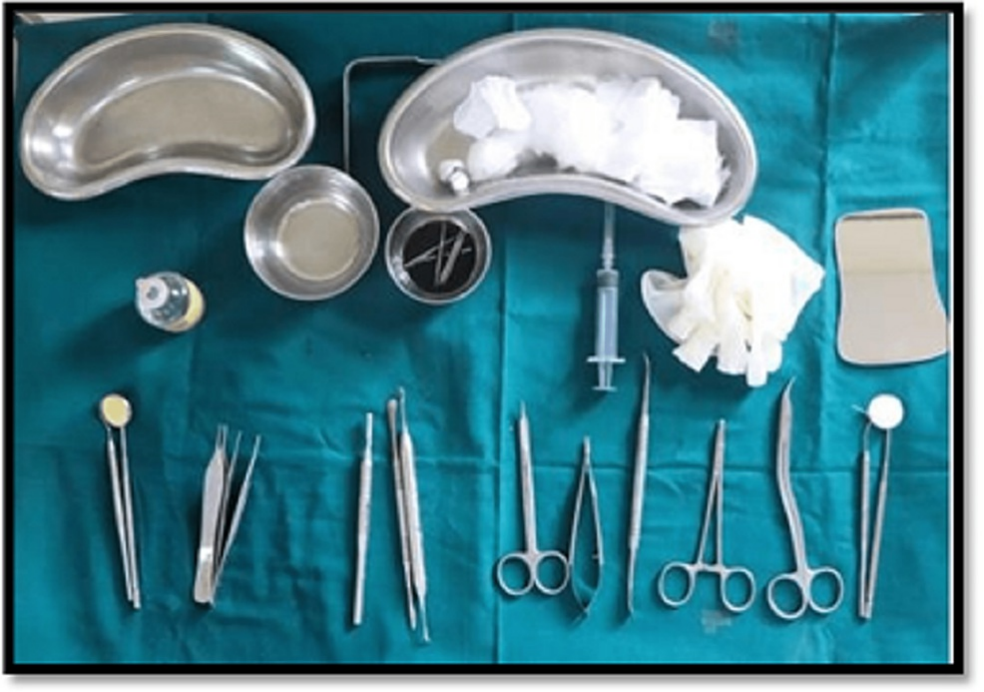
Armamentarium

Armamentarium for corticotomy (Figure 1) periosteal elevator, S\scalpel, B P blade and handle, burs - round no.2 and no.3 and straight bur no.702, Micromotor handpiece, Local anesthesia (2% lignocaine and 1:8000 adrenaline), Syringe - 2 ml for lignocaine, 10 ml for irrigation.

Armamentarium for the orthodontic procedure: Mclaughlin Bennet and Trevisi (MBT) 0.22-inch slot extraction kit, Nitinol coil springs (JJ Orthodontics Private Limited), Dontrix gauge (JJ Orthodontics Private Limited)

Armamentarium for root resorption analysis: 3-D Cone Beam computed Tomography (CBCT) was performed at 80kV, 5mA, 7.5-second exposure, and a 10*10 field of view J-Morrita), pre-treatment and post-treatment CBCT of the subjects in DICOM format, RealGuide 5.0 software for DICOM to stereolithography (STL) format conversion, Solidworks 2021 software for CBCT analysis.

A split-mouth study design was used where one side of the arch was designated as the vertical technique and the contra lateral as the Indentation technique. The side was allotted randomly using a simple randomization technique. The side was allotted randomly using a simple randomization technique wherein chits were picked up in the department by a person who is not an orthodontist by profession. Two chits consisting of vertical and indentation names and 16 chits containing sample numbers from one to 16 were allocated for randomization for the split-mouth design. Based on the above method of allocation samples were divided further into these four groups. 1. Maxillary vertical 2. Maxillary indentation 3. Mandibular vertical 4. Mandibular indentation

**Figure 2:**
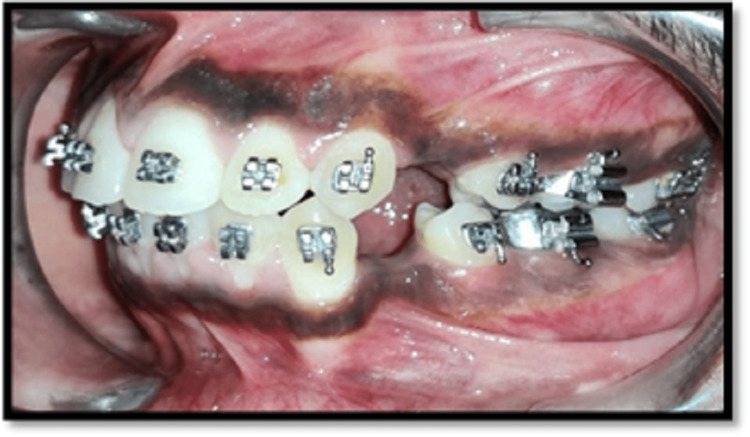
Right lateral view after alignment

**Figure 3:**
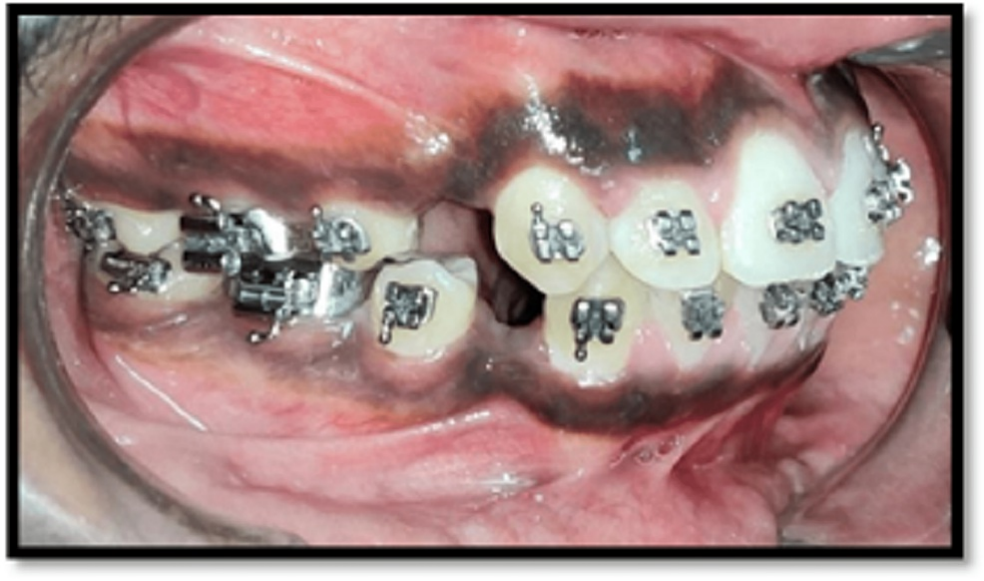
Left lateral view after alignment

**Figure 4:**
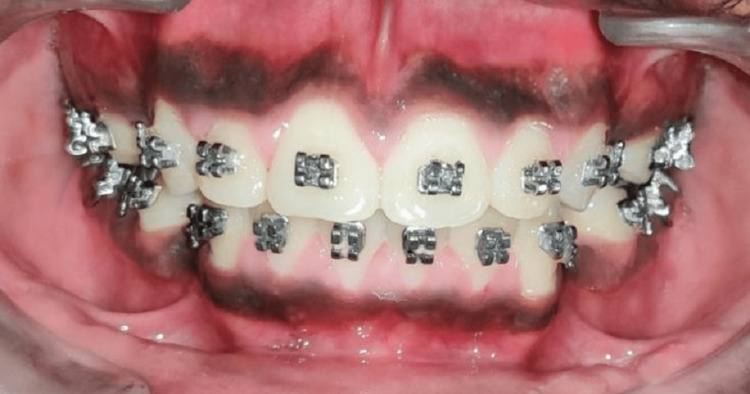
Frontal view after alignment

**Figure 5:**
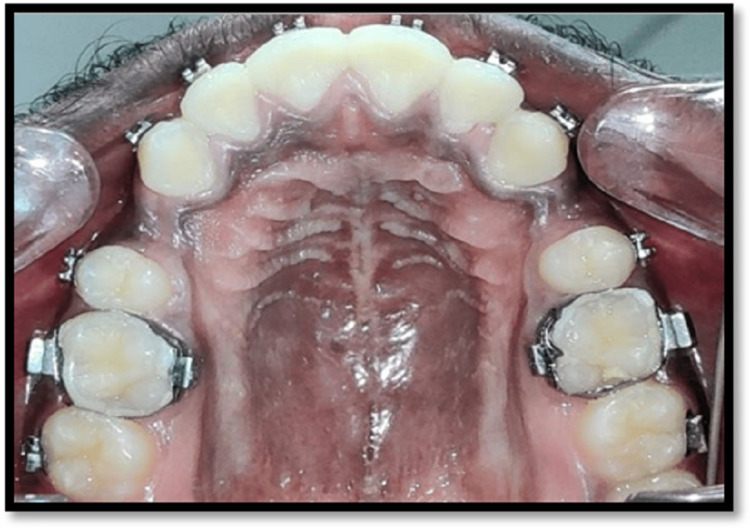
Maxilla

**Figure 6:**
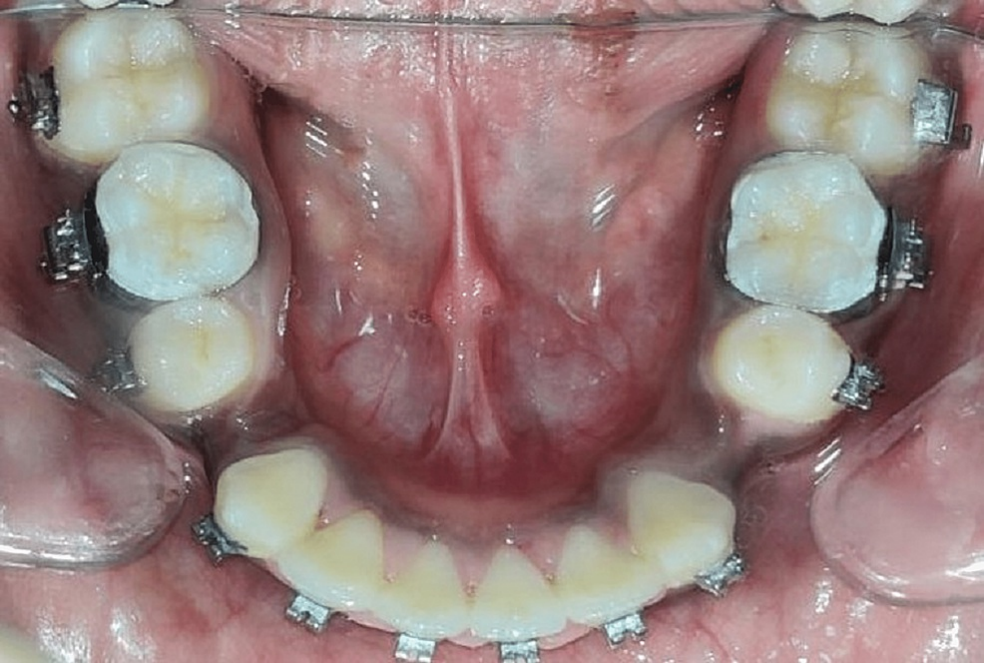
Mandibular occlusal view

The leveling and aligning were initiated until a level slot was achieved along with bite correction. This was further examined by checking for root parallelism using a radiographic aid. This also further helped in placing the corticotomy cuts during the procedure (Figures 2, 3, 4, 5, 6) 

The extraction of the premolar was performed just before the retraction was started with the intention of inducing RAP phenomenon through corticotomy. The patients were subjected to corticotomy procedure immediately after the extraction of the premolar teeth. A CBCT (T1) was carried out before the retraction stage was started. The procedure was carried out by a single experienced specialist (periodontology and implantologist). The entire procedure was performed under local anesthesia.

**Figure 7:**
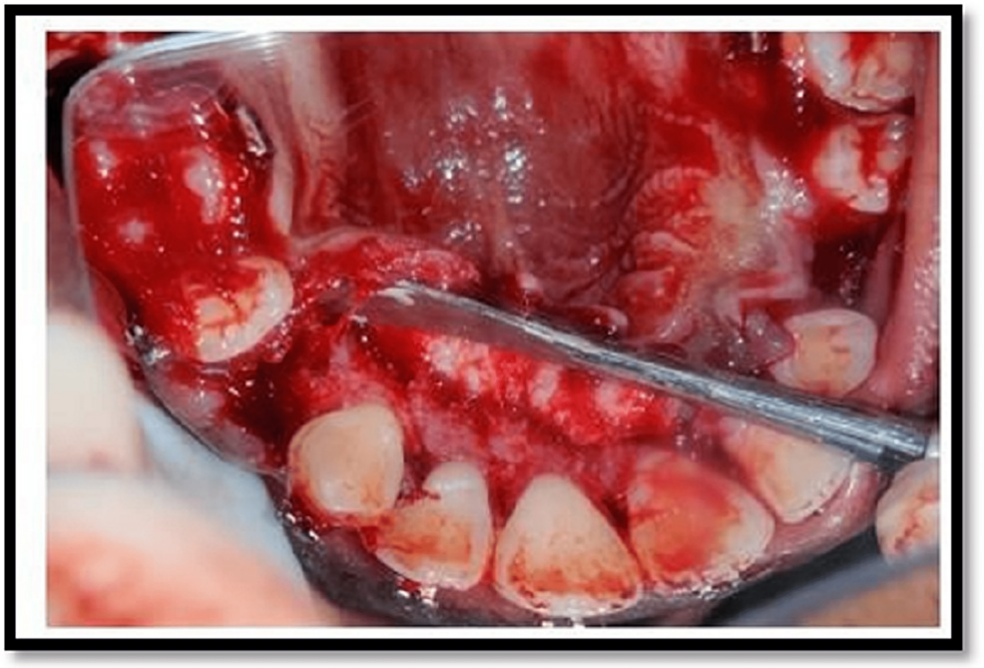
Corticotomy cuts on palatal aspect

**Figure 8:**
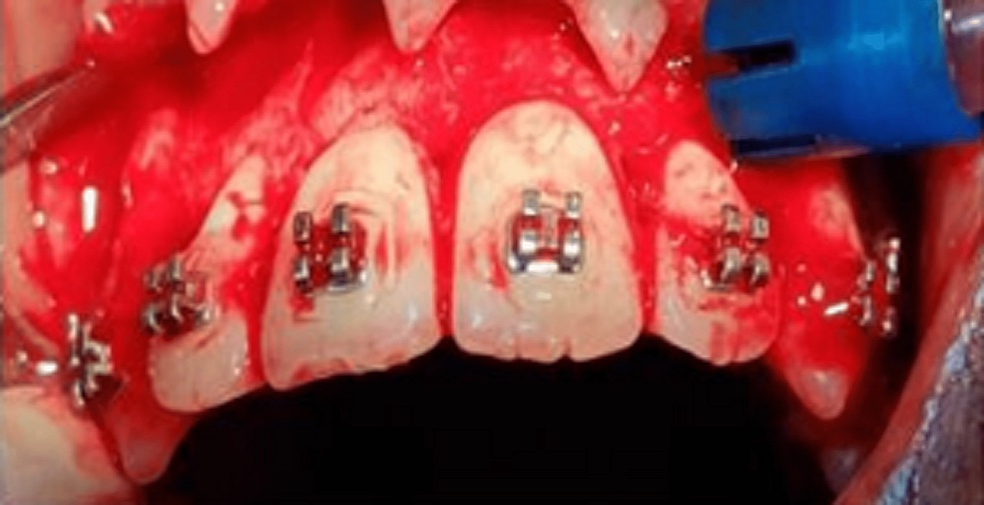
Corticotomy cuts on buccal aspect

**Figure 9:**
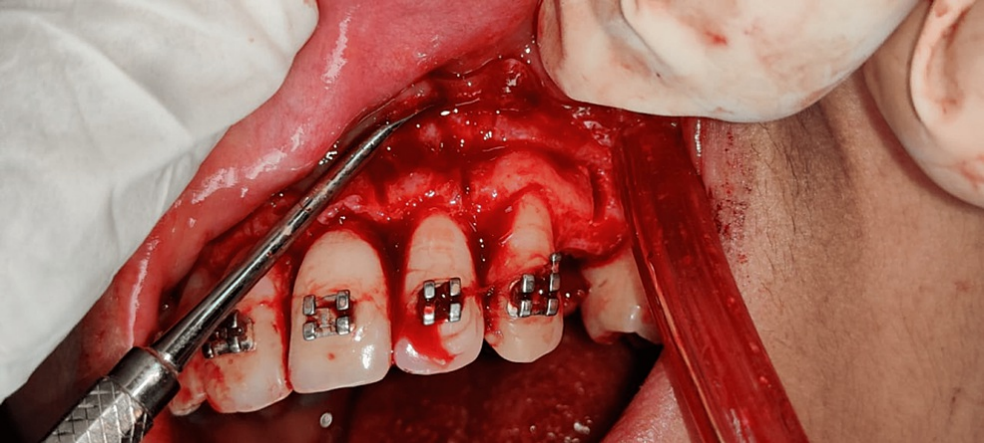
Vertical corticotomy cuts

The mucogingival flaps were elevated on the labial side and lingual side to completely expose the cortical bone. Indentation corticotomy was performed with bur no.2 and 3 respectively as per the requirement and vertical cuts were performed with bur no. 702. Cuts were restricted to the cortical plate and a depth of about 1.5 mm cuts was placed. The cuts were placed on both the labial and palatal aspects at the extraction site in the maxilla and on the labial and lingual aspects of the mandible. The orthodontic force loading was done on the same day of the procedure and a retraction force of 130 to 140 gm was measured using a Dontrix gauge and applied using a NiTi close coil spring of length 9 mm or 12 mm as required for the activation. The immediate loading of the orthodontic force was done so as to utilize the regional acceleratory phenomenon completely as the RAP phenomenon is induced immediately and peaks after 10-14 days (Figures 7, 8, 9).

The patients were recalled after seven days for suture removal after which the patients were recalled once every 21-25 days for follow-up and re-activation of the Niti closed coil springs (four to six months). The follow-ups were done until the space closure occurred following which a second CBCT (T2) was taken following which the analysis for root resorption was done.

**Figure 10:**
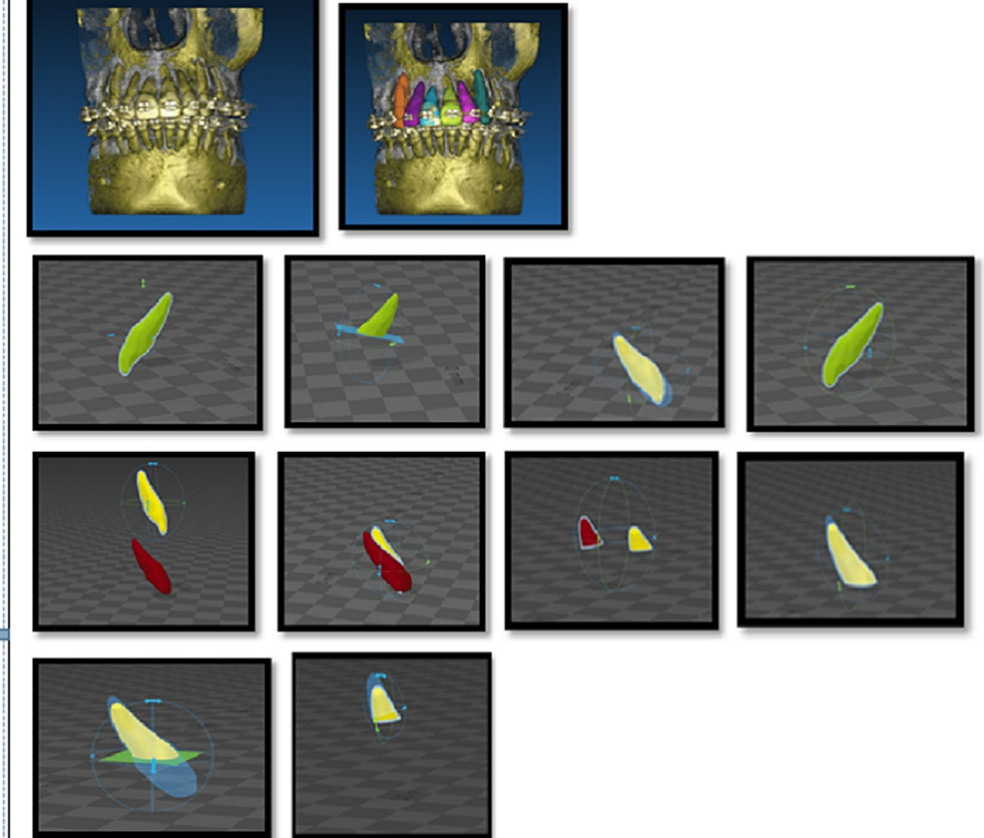
Steps in the linear and volumetric analysis of measuring the external apical root resorption

The CBCT of the patients was taken, one before retraction just before the corticotomy procedure, and the second CBCT (T2) taken after the space closure had occurred. These CBCTs were then evaluated for the total root resorption that had occurred during the space closure phase. Mean and SD was calculated for the amount of root resorption pretreatment and post-treatment (Figure 10).

Statistical Analysis

Inferential Statistics

Inter-group comparison of the root resorption was done by paired t-test. Paired t-test or the dependent sample t-test was the statistical test that was used to determine the mean difference between the two groups assessing the amount of root resorption between the two groups of corticotomy procedures. The level of significance was set at P<0.05. The minimum Sample size required for this study is 16 subjects. The sample size has been estimated using the GPower software v.3.1.9,2. Since it is a follow-up study for four to six months with a repeat radiograph, there is a chance for dropouts the sample was raised to 20.

## Results

The results are based on 16 samples of central incisors, lateral incisors, and canines whose maxillary and mandibular data were collected as per randomly divided quadrants into vertical and indentation groups.

The following results can be tabulated,

Comparing the amount of root resorption between the maxillary and mandibular central incisors, lateral incisors and canines in the two different techniques namely indentation and vertical cut techniques (linear and volumetric)

Among the maxillary anterior teeth, the lateral incisors showed the maximum amount of root resorption according to the linear analysis. In the indentation technique, a mean value of 0.6313 was obtained and in the vertical technique, a mean value of 0.8875 was seen.

According to the volumetric analysis, the maxillary canines showed the maximum amount of root resorption. In the indentation technique there was a mean root resorption of 1.1188 and the vertical technique showed a mean value of 1.1188.

In the mandibular arch among the anterior teeth, the canine underwent the maximum amount of resorption according to both the linear and volumetric analysis. In the linear measurements, a mean root resorption of 0.6688 was seen in the indentation technique, and in the vertical technique, a mean root resorption of 0.6050 was seen.

In the volumetric analysis, in the indentation technique, a mean root resorption of 1.5125 was seen and in the vertical technique, a mean root resorption of 1.2750 was seen.

Comparing the amount of root resorption between the two techniques namely indentation corticotomy and vertical corticotomy in the maxillary and mandibular anterior teeth.

When compared to the amount of root resorption between the two techniques, among the anterior teeth, the lesser amount of root resorption occurred in the indentation corticotomy technique as compared to the vertical corticotomy technique (Tables 1, 2, 3, 4).

**Table 1:** Mean difference in the pre and post root length between indentation and vertical corticotomy techniques between central incisors, lateral incisors, canines - Maxilla SD: standard deviation

Technique	Maxillary central incisor		Maxillary Lateral incisors		Maxillary Canine	
	Pre linear(mm) Mean ± SD	Post linear (mm) Mean ± SD	Pre linear(mm) Mean ± SD	Post linear (mm) Mean ± SD	Pre linear (mm) Mean ± SD	Post linear (mm) Mean ± SD
Indentation	20.0±5.4	19.6±5.3	19.3±5.3	18.6±5.1	21.2±5.9	20.8±5.8
Vertical	20.4±5.5	19.9±5.4	19.1±5.2	20.0±8.9	21.0±5.9	20.4±5.7
p value	0.861	0.902	0.903	0.598	0.934	0.830

**Table 2:** Mean difference in the pre and post root volume between indentation and vertical corticotomy techniques between central incisors, lateral incisors, canines - Maxilla SD: standard deviation cumm: cubicmillimeter

Technique	Maxillary central incisor		Maxillary Lateral incisors		Maxillary Canine	
	Pre volume(cumm) Mean ± SD	Post volume(cumm) Mean ± SD	Pre volume (cumm) Mean ± SD	Post volume(cumm) Mean ± SD	Pre volume(cumm) Mean ± SD	Post volume(cumm) Mean ± SD
Indentation	48.9±13.6	48.1±13.3	46.9±12.8	46.0±12.6	48.7±14.0	47.5±14.1
Vertical	47.1±12.8	46.1±12.6	42.3±12.1	40.6±12.1	44.6±12.6	43.3±12.3
p value	0.702	0.668	0.304	0.230	0.398	0.368

**Table 3:** Mean difference in the pre and post root length between indentation and vertical corticotomy techniques between central incisors, lateral incisors, canines - Mandible SD: standard deviation

Technique	Mandibular Central incisor		Mandibular Lateral incisors		Mandibular Canine	
	Pre linear(mm) Mean ± SD	Post linear (mm) Mean ± SD	Pre linear (mm) Mean ± SD	Post linear (mm) Mean ± SD	Pre linear (mm) Mean ± SD	Post linear (mm) Mean ± SD
	Indentation	15.2±6.2	14.8±6.0	14.3±5.6	14.0±5.6	18.3±7.2	17.6±6.9
Vertical	14.3±5.7	14.0±5.5	15.1±6.1	14.7±6.0	18.4±7.2	17.7±6.9
p value	0.675	0.690	0.700	0.718	0.992	0.994

**Table 4:** Mean difference in the pre and post root volume between indentation and vertical corticotomy techniques between central incisors, lateral incisors, canines - Mandible SD: standard deviation cumm: cubicmillimeter

Technique	Mandibular Central incisor		Mandibular Lateral incisors		Mandibular Canine	
	Pre volume(cumm) Mean ± SD	Post volume(cumm) Mean ± SD	Pre volume(cumm) Mean ± SD	Post volume(cumm) Mean ± SD	Pre volume (cumm) Mean ± SD	Post volume(cumm) Mean ± SD
Indentation	31.9±13.1	30.9±12.6	30.6±12.0	29.9±11.7	31.6±12.8	30.1±12.2
Vertical	30.8±12.2	30.1±11.9	31.5±12.7	29.7±12.0	30.9±12.5	29.6±11.9
p value	0.811	0.862	0.838	0.961	0.873	0.910

## Discussion

Orthodontic treatment is frequently related to the occurrence of apical root resorption. The concentration of orthodontic forces on the root, especially on the apex, can cause biological changes in the cementum and periodontal ligament, resulting in root resorption. In 1998, Brezniak et al. presented a comprehensive review of the literature on root resorption and considered several biological, mechanical, and clinical factors. Risk factors for external apical root resorption (EARR) can be categorized as patient-related and treatment-related. Patient-related factors include; genetics, systemic factors, asthma and allergies, chronic alcoholism, the severity of malocclusion, tooth-root morphology, a previous history of root resorption, alveolar bone density, root proximity to cortical bone, endodontic treatment, and patient age and sex. Orthodontic treatment-related risk factors include; the treatment duration, magnitude of applied force, direction of tooth movement, amount of apical displacement, and method of the force application. Most studies concluded that the amount of resorption might not be anticipated with certainty based on any of the reported etiologic factors [6]. The literature suggests that regional acceleration of bone remodeling following corticotomy procedure with the intention of accelerating orthodontic tooth movement is a special phenomenon. The moderate orthodontic load-induced sterile inflammatory process ordinarily associated with orthodontic tooth movement is superimposed on the selective decorticotomy-induced sequence of bone remodeling, resulting in accelerated tooth movement. Following surgically induced insult to cortical bone, bone turnover begins within a few days, peaks at 1 to 2 months after surgery, and subsides within six months when healing is complete. In the context of corticotomy-facilitated orthodontics, the process of bone healing and remodeling lasts approximately four to six months; accelerated orthodontic tooth movement can only occur during this period [6-7]. The results of a study by Acar et. al showed that the application of discontinuous force results in less root resorption than the application of continuous force [8].

Compared with conventional orthodontic treatment, corticotomy-facilitated orthodontics is reportedly associated with diminished frequency and magnitude of external apical root resorption. This may be related to the reduced formation of necrotic and hyalinized tissues at the same time rapid removal of the same thereby contributing to the overall decrease in the length of active orthodontic treatment. Increased tissue turnover commensurate with RAP and diminished bone density would logically favor less root resorption. A study by Eatemad in 2012 concluded that patients treated with conventional orthodontics showed higher rates of apical root resorption than patients treated with corticotomy-facilitated orthodontics [7]. These results were in agreement with studies done by Germec et al. in 2006, in which they agreed that there is always less apical root resorption in corticotomy cases [9]. Bone turnover is well known to be accelerated after a bone fracture, osteotomy, or bone grafting. This could be explained by a regional acceleratory phenomenon (RAP); i.e., osteoclasts and osteoblasts increase by local multicellular mediator mechanisms containing precursors, supporting cells, blood capillaries, and lymph. RAP also occurs in the mandible. Similarly, bone turnover is increased by RAP after a corticotomy [10]. 

Wilcko et al. (2005) published various case reports on corticotomy and concluded that there is less root resorption due to decreased resistance of cortical bone [11]. The split-mouth study by Aboalnaga et al.,(2019) also found no significant difference in EARR in the four-month canine retraction period. The study used cone-beam computed tomography (CBCT) for the measurement of root resorption [12]. 

In 2015, Alikhani et al. studied MOPs as a minimally invasive accelerated technique and found that external apical Root Resorption did not increase following MOP treatment [13]. Darendeliler et al. (2021) studied the effect of Peizoincision (PzC) on Root Resorption associated with an orthodontic force of 150g using computed tomography and found that the PzC procedure resulted in an increase in Root Resorption on all surfaces and vertical thirds when compared with control sides after application of orthodontic force for 28 days. However, only total Root Resorption values reached statistical significance. It was found that the PzC procedure resulted in a 44% average increase in RR compared with the control side [14].

Corticotomy facilitated technique was used in our study as documented evidence proved it to be a safe modality for intervention in reducing the amount of root resorption and accelerating the overall treatment duration. Comparison of the root resorption between the Indentation and vertical corticotomy techniques was the purpose of this study.

In this present study, the amount of root resorption that occurred in the indentation corticotomy techniques was less compared to the vertical corticotomy techniques in both the linear and the volumetric analysis. But this was not statistically significant because the amount of resorption can be affected by the shape, size, and number of the corticotomy cuts as well. The perforation design will lead to lesser harm to the root structures and can be placed with better ease when there is a reduced spacing between the teeth roots due to which there was a reduced amount of root resorption in the indentation corticotomy technique. Hence it was observed from our study that indentation corticotomy cuts for accelerated tooth movement are considered to be more safe, effective method, lesser invasive to the surrounding tissues, technique sensitive, and good RAP Phenomenon, with a Rapid healing process compared to the vertical corticotomy technique.

Comparing the amount of root resorption between the two arches namely the maxillary and mandibular arch between the two different corticotomy techniques namely indentation corticotomy and vertical corticotomy in the maxillary and mandibular anterior teeth. In our study, we saw that the maxillary arch showed higher amounts of root resorption in both indentation and vertical corticotomy techniques. This was true in both linear as well as volumetric analyses. 

Limitations

long term studies are needed to check the stability of treatment results. Due to Covid -19 pandemic lockdowns the follow-up appointment was rescheduled. Few breakages in the appliance were noted in the rescheduled appointment.

Scope for future study

The long-term effects and the stability of the treatment following these corticotomy techniques can be studied. The root resorption occurring during the overall treatment can also be assessed using the three-dimensional radiographic aid.

## Conclusions

The post-space closure results showed root resorption occurred in both techniques ie. Indentation and vertical corticotomy technique in the maxillary and mandibular arch among all the anterior teeth. Among the maxillary anterior teeth, the lateral incisors showed the maximum amount of root resorption according to the linear analysis. In the indentation technique, a mean value of 0.6313 was obtained and in the vertical technique, a mean value of 0.8875 was seen.

According to the volumetric analysis, the maxillary canines showed the maximum amount of root resorption. In the indentation technique there was a mean root resorption of 1.1188 and the vertical technique showed a mean value of 1.1188. In the mandibular arch among the anterior teeth, the canine underwent the maximum amount of resorption according to both the linear and volumetric analysis. In the linear measurements, a mean root resorption of 0.6688 was seen in the indentation technique, and in the vertical technique, a mean root resorption of 0.6050 was seen.

In the volumetric analysis, in the indentation technique, a mean root resorption of 1.5125 was seen and in the vertical technique, a mean root resorption of 1.2750 was seen. When compared to the amount of root resorption between the two techniques, among the anterior teeth, a lesser amount of root resorption occurred in the indentation corticotomy technique as compared to the vertical corticotomy technique. Hence it was observed from our study that indentation corticotomy cuts for accelerated tooth movement are considered to be a more safe, effective method, lesser invasive to the surrounding tissues, technique sensitive, good RAP Phenomenon, with a Rapid healing process compared to the vertical corticotomy technique.
